# Editorials

**Published:** 1896-07

**Authors:** 


THE
### HOMŒOPATHIC PHYSICIAN,

A MONTHLY JOURNAL OF
HOMŒOPATHIC MATERIA MEDICA AND CLINICAL MEDICINE.

"If our school ever gives up the strict inductive method of Hahnemann, we are lost, and deserve only to be mentioned as a caricature in the history of medicine."—CONSTANTINE HERING.

Vol. XVI. JULY, 1896. No. 7.

### EDITORIALS.

MERCURIUS-VIVUS.—This remedy has discharge of blood before, during, and after stool. This is similar to Lycopodium. There is a discharge of mucus from the rectum which is similar to Apis. Those who wish to know all about the indications for remedies under the symptom of mucous discharge from the rectum should study *Bell On Diarrhœa.*

Rectum black, discharging blood, is an indication for Mercurius. If there be at the same time burning pains we should think of Arsenicum.

Under Mercury the urine may be in great abundance and very pale. This also is true of Lycopodium. Rhus-toxicodendron has scanty urine. The urine smells sour and pungent. This indicates both Mercurius and Nitric-acid. Hemorrhage from the urethra indicates Mercurius and Cactus-grandifloris. Mercurius has liver-spots on the genitals.

Mercurius is the most important remedy for chancres on the prepuce and glans. Dr. Lippe gave the following indications for chancres in the course of his remarks upon Mercury.

Corallium: chancres are exceedingly painful and sensitive to contact. The patient does not want them touched. The discharge from them is thin, offensive, and black.

Hepar: ulcers on the prepuce with corrosive pains, also burning and stinging and extremely sensitive to contact.

Kali-bichromicum: ulcers become deeper without spreading. They look as if cut out with a punch.

Sulphur: ulcers on the glans, sensitive to touch.

Mercurius-jodatus-ruber: the ulcers are almost painless.

Thuja: red, black, dirty ulcers upon the glans.

Silicea: ulcers upon the glans with much destruction of tissue and generally a watery discharge.

Nitric-acid: deep ulcers on the glans with indurated edges.

Mercurius is strongly indicated in phimosis and paraphimosis.

Mercurius has profuse menstruation, like Calcarea-carbonica.

Mercurius has acrid greenish leucorrhœa. It indicates the probable presence of gonorrhœa.

Mercurius has cough and hoarseness. There is no expectoration at night.

Mercurious and Nitric-acid are both indicated in violent catarrhs with cough, hoarseness, coryza, and sore throat.

Natrum-muriaticum has violent racking cough.

The cough of Mercury is aggravated from lying on the left side. This is similar to Phosphorus.

The Mercury cough is also worse from walking, running, and during sleep.

Mercurius has shortness of breath when ascending stairs, like Calcarea-carbonica.

Mercurius has inflammation of the lungs with stitches in the right side of the chest through from the shoulder blade. This symptom induced Dr. Lippe to remark that stitches in right side of the chest, upper third, indicate Borax. Stitches in the lower third of right side of chest indicate Bryonia. Stitches in upper part of left chest indicate Phosphorus. Stitches in lower part of left chest indicate Sulphur.

Thus upon a diagram of the chest we might write the names of five remedies above mentioned, thus: above right nipple, Borax and Mercury; below right nipple, Bryonia; above left nipple, Phosphorus, and below left nipple, Sulphur.

In this connection Dr. Guernsey's key-note for Arsenicum may be mentioned. Pain in the apex of the right shoulder or all over upper part of right chest and at the same time pain in the left hypochondrium. Thus the chest pains of Arsenicum are diagonal. Apis is another one of Dr. Guernsey's diagonal remedies. It has a key-note, pain in left chest and at the same time in the right ovary.

In 1879 Dr. Lippe, conversing with the editor of this journal upon the foregoing symptoms, spoke of the indications for Mercury in pneumonia as exemplified in a case which he was treating. The principal symptoms were: Chill before the patient went to bed, followed by high fever; pulse 120 and like a hammer; it became lower in the evening; great drowsiness all day; could not keep his eyes open; sleeping all day; skin very sallow; imprints of the teeth upon the tongue; the tongue was swollen and flabby; pain in the chest, right upper lobe of the lungs through to the back; it is a stitch, and is stronger under Mercury than under any other remedy; urine brown and scanty. After a prescription of a high potency of Mercury the patient became better, but profuse perspiration set in, lasting all night. He had a bad cough, which was quite short but persistent. The patient could not speak for coughing. No medicine was given for the cough, as Dr. Lippe considered it to be a necessity, and that it would be hazardous to check it. He regards the character of cough as presaging a favorable termination to the disease. The old school, he further remarked, give Anodyne cough syrups in these cases, suppressing the cough, and the deatn of the patient soon happens. In prescribing for this case, the symptoms not necessarily belonging to the disease, but peculiar to the patient, were used in making the prescription.

---

HERING ON TYPHOID FEVER.—The editor announces that he has begun the publication of the old monograph of *Dr. Hering on Typhoid Fever*, long since out of print. The original publication was a cumbersome quarto. It has been reduced to the size of a duodecimo, so that it is handy for the pocket. The work was undertaken at the request of a number of subscribers, who have frequently expressed a desire to see a new edition of so valuable a book in a more convenient form than the original. It is to be issued as a supplement to THE HOMŒOPATHIC PHYSICIAN.

